# Treatment of advanced gallbladder cancer: A SEER‐based study

**DOI:** 10.1002/cam4.2679

**Published:** 2019-11-13

**Authors:** Weipu Mao, Fang Deng, Dongyan Wang, Li Gao, Xiuquan Shi

**Affiliations:** ^1^ Department of General Surgery The People's Hospital of Yingshang Fuyang China; ^2^ Department of Clinical Laboratory The People's Hospital of Yingshang Fuyang China; ^3^ Department of Gastroenterology Pudong New Area Gongli Hospital Affiliated to Naval Military Medical University Shanghai China; ^4^ Department of Obstetrics and Gynecology The People's Hospital of Yingshang Fuyang China

**Keywords:** advanced gallbladder cancer, chemotherapy, SEER, surgery, survival

## Abstract

**Purpose:**

The treatment of advanced gallbladder cancer (GBC) remains controversial. Therefore, the purpose of this study was to explore treatment choices for advanced GBC.

**Methods:**

We identified four different treatments from the surveillance, epidemiology, and end results (SEER) database: surgery, chemotherapy (CT), surgery and chemotherapy (Surgery + CT), and no surgery/no chemotherapy (No surgery/No CT). Kaplan‐Meier method and Cox proportional hazards regression method were used to determine the risk factors for overall survival (OS) and cancer‐specific survival (CSS). In addition, patients in AJCC stages III and IV stage were matched with 1:1 propensity score matching (PSM) for diagnosis age, race, marital status, histological type, tumor grade, and treatment pattern to decrease the possibility of selection bias.

**Results:**

A total of 288 AJCC stage III patients and 4239 AJCC stage IV patients with advanced GBC were identified from the SEER database between 2004 and 2015. Treatment pattern was an independent risk factor for patients with advanced GBC. For all patient, AJCC stage III patients and AJCC stage IV patients, “Surgery + CT” treatment minimized the OS and CSS in advanced GBC patients. In addition, after the PSM analysis, the “Surgery + CT” treatment still significantly decreased patient OS and CSS.

**Conclusions:**

“Surgery + CT” treatment can provide survival benefits for patients with advanced GBC. In addition, “Surgery + CT” treatment was not fully utilized and may further improve the survival rate of GBC patients.

## Graphical abstract

Surgery and chemotherapy (Surgery + CT) can provide survival benefits for patients with advanced gallbladder cancer (GBC). The treatment of “Surgery + CT” is not fully utilized, and the combined use of “Surgery + CT” can improve the survival rate of GBC patients.

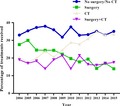

**Abbreviations**
AJCC: American Joint Committee on Cancer
CI: confidence interval
CSS: cancer‐specific survival
CT: chemotherapy
GBC: gallbladder cancer
HR: hazard ratios
OS: overall survival
PSM: propensity score matching
RT: radiotherapy
SEER: surveillance, epidemiology, and end results

## 1 INTRODUCTION

Gallbladder cancer (GBC) is a common malignant tumor in the biliary system, accounting for approximately 2/3 of biliary system tumors, and its incidence is increasing.^1^, ^2^ Although relatively uncommon, it is the sixth common form of digestive system cancer. In 2019, an estimated 12 360 new cases were diagnosed, and 3960 patients died from GBC and other biliary cancer in the United States.^3^ Because of the insidious onset, rapid progression, and early asymptomatic characteristics of GBC, diagnosis is usually not made until intraoperative and postoperative pathological examinations, when the disease is already in moderate and advanced stages, and the therapeutic effect are poor.^4^

The median survival time of patients with GBC is less than 1 year, the overall survival (OS) is approximately 17.8%‐21.7%, and the 5‐year OS is only 5%.^1^, ^5^ GBC treatments include surgery, chemotherapy (CT), radiotherapy (RT), and other immunotherapy.^6^, ^7^, ^8^ Although GBC has high invasiveness and metastasis, surgical resection remains recognized as the best treatment.^9^, ^10^ The 5‐year survival rate of early T1 GBC patients is as high as 95%‐100%. However, for patients with T3 stage and T4 stage, the 5‐year survival rate is only 23% and 12%, respectively.^11^

Both in China and abroad, most diagnosed GBC patients are in moderate and advanced stages. The treatment of GBC still confuses many physicians, even experienced surgeons. The treatment of patients with advanced GBC remains especially controversial. In our study, we used data from the surveillance, epidemiology, and end results (SEER) cancer registration database to explore the treatment options for patients with advanced GBC.

## 2 PATIENTS AND METHODS

### 2.1 Patients selection

The SEER database is currently the largest publicly available cancer database, covering approximately 28% of the US population.^12^ All cases are from the SEER Program (http://www.seer.cancer.gov) SEER*Stat database released in November 2017: version 8.3.5; SEER 18 Regs Custom Data (with additional treatment field), Nov 2017 Sub (1973‐2015 varying) database. The SEER database contains information about patient demographics and cancer characteristics, such as sex, age at diagnosis, year of diagnosis, race, marital status, tumor grade and stage, histological type, treatment, and patient survival time.

Using the "Primary Site—labeled" variable, we selected tumor cases from the primary site of the gallbladder diagnosed between 2004 and 2015. The study included only patients with American Joint Committee on Cancer (AJCC) stage III and IV cancer. According to the 2004 AJCC staging principle, stage III is defined as “T4M0, any N,” and stage IV is defined as “M1, any T or any N.” Only patients above 18 years of age were included in this study. Patients with any of the following criteria were also excluded: unknown treatment, not the first tumor, unknown survival time, and unknown marital status. Finally, 4527 eligible patients diagnosed with GBC remained.

### 2.2 Study variables

Definition and information about the variables of sex, diagnosis age, year of diagnosis, race, marital status, tumor grade, histological type, and survival time can be found in the SEER database. OS and cancer‐specific survival (CSS) were the primary study endpoints. For OS, death from any cause was considered as an event, and the survivor was regarded as censored. For the CSS analysis, deaths caused by GBC were considered events, and deaths from other causes or survivors were censored.

For the diagnosis age, we divided all patients into three groups: less than 60 years old, 60‐80 years old, and older than 80 years old.

For marital status, patients were divided into a Married group, an Unmarried group, and an Unknown marital status group. Unmarried patients include Single, Separated, Divorced, and Widowed.

For race, patients were divided into a Non‐Hispanic White group, a Non‐Hispanic Black group, a Hispanic group, and an Others group.

The ICD‐0‐3 site/histology validation list was used to distinguish adenocarcinoma, squamous cell carcinoma, and other histological types.

Grade was defined by the following codes: well differentiated (Grade I); moderately differentiated (Grade II); poorly differentiated (Grade III); undifferentiated (Grade IV); and unknown grade.

### 2.3 Statistical analysis

Pearson's chi‐square analysis was used to analyze and evaluate the different clinical characteristics between different treatment patterns. The Kaplan‐Meier curve was used to estimate the OS and CSS in different groups, and the differences between the curves were analyzed by log‐rank test. Univariate and multivariate Cox regression models were performed to estimate the hazard ratios (HR) and 95% confidence interval (CI) to analyze the independent prognostic factors associated with OS and CSS in GBC patients.

According to AJCC stage, the patients were divided into AJCC stage III and IV groups. 1:1 propensity score matching (PSM) was to reduce the selection bia of the two groups of baseline variables, including age, race, marital status, histological type, grade, and treatment pattern seven variables. After PSM, the clinicopathological features of the patients were reevaluated according to AJCC stage. All statistical analyses were conducted with the Statistical Package for the Social Sciences software (version 24.0; IBM Corporation). A *P* value ≤ .05 was considered statistically significant.

## 3 RESULTS

### 3.1 Demographic and clinical characteristics

From 2004 to 2015, our study cohort included a total of 4527 eligible GBC patients. Among them, 1575 patients with “No surgery/No CT,” 938 patients with “Surgery”, 1222 patients with “CT” and 792 patients with “Surgery + CT”. The demographic and clinical characteristics of GBC patients with different treatment patterns are shown in Table 1. Male patients accounted for 30.7%, and female patients accounted for 69.3%. Moreover, there were 288 patients with AJCC stage III and 4239 with AJCC stage IV. Chi‐square test showed significant differences in some variables and treatment patterns, including diagnosis age, sex, race, marital status, histological type, tumor grade, and AJCC stage (All *P* < .05).

**Table 1:** Characteristics for different metastasis in our study

Characteristic	Total	No surgery/No CT	Surgery	CT	Surgery + CT	*P* value
	n = 4527	n = 1575 (%)	n = 938 (%)	n = 1222 (%)	n = 792 (%)	
Sex						
Female	1391	509 (32.3)	272 (29.0)	403 (33.0)	207 (26.1)	.003
Male	3136	1066 (67.7)	666 (71.0)	819 (67.0)	585 (73.9)	
Age at diagnosis						
<60 y	1057	210 (13.3)	164 (17.5)	405 (33.1)	278 (35.1)	<.001
60‐80 y	2551	825 (52.4)	540 (57.6)	718 (58.8)	468 (59.1)	
>80 y	919	540 (34.3)	234 (24.9)	99 (8.1)	46 (5.8)	
Race						
Non‐Hispanic White	2543	873 (55.4)	530 (56.5)	689 (56.4)	451 (56.9)	.007
Non‐Hispanic Black	580	190 (12.1)	99 (10.6)	189 (15.5)	102 (12.9)	
Hispanic	922	329 (20.9)	216 (23.0)	214 (17.5)	163 (20.6)	
Others	482	183 (11.6)	93 (9.9)	130 (10.6)	76 (9.6)	
Marital status						
Married	2352	651 (41.3)	473 (50.4)	741 (60.6)	487 (61.8)	<.001
Divorced/separated	483	159 (10.1)	88 (9.4)	145 (11.9)	91 (11.5)	
Windowed	1066	516 (32.8)	259 (27.6)	185 (15.1)	106 (13.4)	
Single	626	249 (15.8)	118 (12.6)	151 (12.4)	108 (13.6)	
Histological type						
Adenocarcinoma	3278	923 (58.6)	773 (82.4)	914 (74.8)	668 (84.3)	<.001
Squamous cell carcinoma	167	44 (2.8)	54 (5.8)	31 (2.5)	38 (4.8)	
Others	1082	608 (38.6)	111 (11.8)	277 (22.7)	86 (10.9)	
Grade						
Grade I	157	20 (1.3)	62 (6.6)	23 (1.9)	52 (6.6)	<.001
Grade II	804	105 (6.7)	281 (30.0)	121 (9.9)	297 (37.5)	
Grade III	1219	221 (14.0)	477 (50.9)	184 (15.1)	337 (42.6)	
Grade IV	86	15 (1.0)	35 (3.7)	12 (1.0)	24 (3.0)	
Unknown	2261	1214 (77.1)	83 (8.8)	882 (72.2)	82 (10.4)	
AJCC stage						
III	288	84 (5.3)	72 (7.7)	56 (4.6)	76 (9.6)	<.001
IV	4239	1491 (94.7)	866 (92.3)	1166 (95.4)	716 (90.4)	

Note

Percentages may not total 100 because of rounding.

Abbreviations: AJCC, American Joint Committee on Cancer; CT, chemotherapy; Grade I, well differentiated; Grade II, moderately differentiated; Grade III, poorly differentiated; Grade IV, undifferentiated.

### 3.2 Trend in different treatment patterns

**Figure 1:**
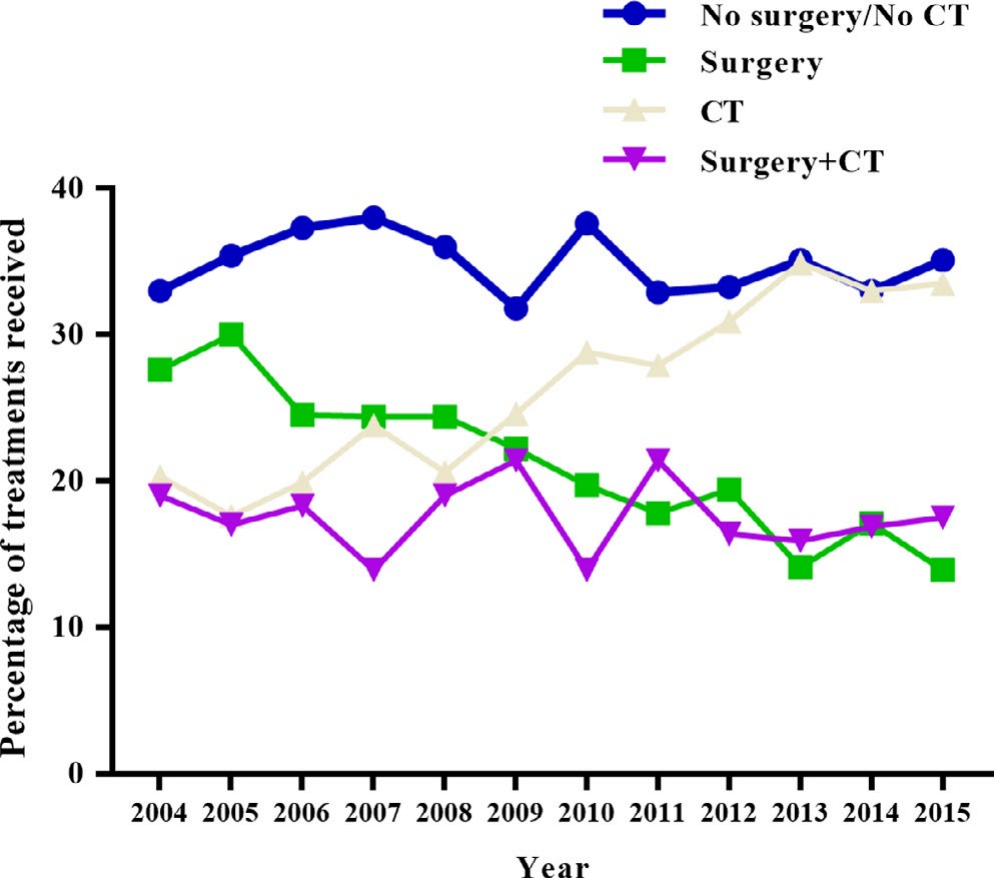
Trends in the proportion of four treatment modes in patients with advanced gallbladder cancer between 2004 and 2015

As shown in Table 2, the proportion of patients who accepted the "No surgery/No CT" or "Surgery + CT" models remained relatively stable between 2004 and 2015. Simultaneously, the number and proportion of patients receiving the "CT" model increased each year. The proportion of the "Surgery" treatment was significantly lower in patients with advanced GBC compared with the increase in "CT" mode (Figure 1).

**Table 2:** Changes in the number and proportion of the four treatment methods between 2004 and 2015

Characteristic	Total	No surgery/No CT	Surgery	CT	Surgery + CT	*P* value
	n = 4527	n = 1575 (%)	n = 938 (%)	n = 1222 (%)	n = 792 (%)	
Year of diagnosis						
2004	315	104 (33.0)	87 (27.6)	64 (20.3)	60 (19.0)	<.001
2005	347	123 (35.4)	104 (30.0)	61 (17.6)	59 (17.0)	
2006	322	120 (37.3)	79 (8.4)	64 (19.9)	59 (18.3)	
2007	332	126 (38.0)	81 (24.4)	79 (23.8)	46 (13.9)	
2008	311	112 (36.0)	76 (24.4)	64 (20.6)	59 (19.0)	
2009	374	119 (31.8)	83 (22.2)	92 (24.6)	80 (21.4)	
2010	396	149 (37.6)	78 (19.7)	114 (28.8)	55 (13.9)	
2011	398	131 (32.9)	71 (17.8)	111 (27.9)	85 (21.4)	
2012	427	142 (33.3)	83 (19.4)	132 (30.9)	70 (16.4)	
2013	439	154 (35.1)	62 (14.1)	153 (34.9)	70 (15.9)	
2014	421	139 (33.0)	72 (17.1)	139 (33.0)	71 (16.9)	
2015	445	156 (35.1)	62 (13.9)	149 (33.5)	78 (17.5)	

Note

Percentages may not total 100 because of rounding.

Abbreviation: CT, chemotherapy.

### 3.3 Identification of prognostic factors of OS and CSS in patients with advanced GBC

**Figure 2:**
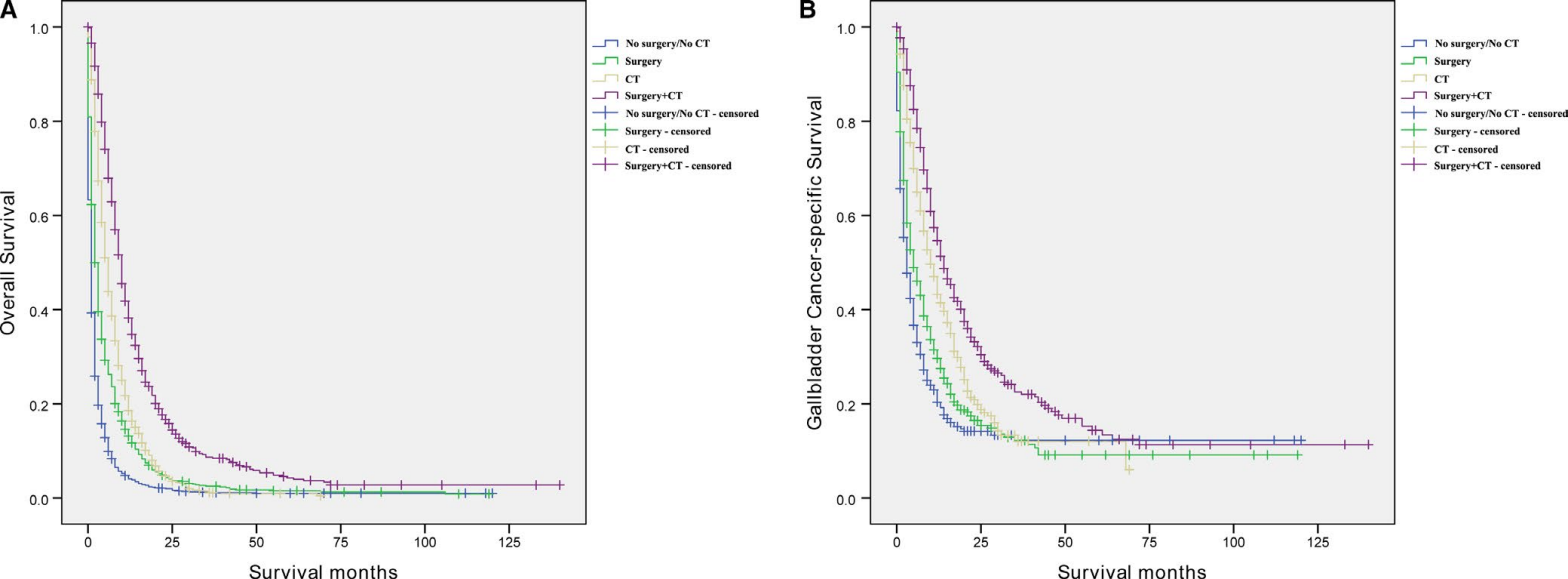
Overall survival and cancer‐specific survival curves of advanced gallbladder cancer patients. A, Overall survival; B, Cancer‐specific survival

Univariate and multivariate Cox regression were used to analyze the factors associated with OS and CSS in patients with advanced GBC. Before matching, as shown in Table 3, the age at diagnosis, histological type, tumor grade, AJCC stage, and treatment pattern affected the OS and CSS in patients with advanced GBC. Multivariate Cox regression showed that “surgery” (vs “No surgery/No CT”; HR = 0.60, 95% CI 0.55‐0.66, *P* < .001), “CT” (vs “No surgery/No CT”; HR = 0.44, 95% CI 0.40‐0.48, *P* < .001), and “Surgery + CT” (vs “No surgery/No CT”; HR = 0.29, 95% CI 0.26‐0.33, *P* < .001) were associated with OS (Figure 2A). Similarly, in terms of CSS, multivariate Cox regression analysis also indicated that treatment pattern was a prognostic factor for patients with advanced GBC (“surgery” vs “No surgery/No CT”; HR = 0.65, 95% CI 0.57‐0.74, *P* < .001; “CT” vs “No surgery/No CT”; HR = 0.44, 95% CI 0.39‐0.50, *P* < .001; “Surgery + CT” vs “No surgery/No CT”; HR = 0.33, 95% CI 0.29‐0.39, *P* < .001) (Figure 2B).

**Table 3:** Univariate and multivariate analysis of OS and CSS rates before propensity score matching

Characteristic	OS				CSS			
	Univariate analysis		Multivariate analysis^a^		Univariate analysis		Multivariate analysis^b^	
	Hazard ratio (95% CI)	*P* value	Hazard ratio (95% CI)	*P* value	Hazard ratio (95% CI)	*P* value	Hazard ratio (95% CI)	*P* value
Sex								
Female	Reference		Reference		Reference			
Male	0.94 (0.88‐1.00)	.049	—	.243	1.02 (0.94‐1.12)	.624		
Age at diagnosis								
<60 y	Reference		Reference		Reference		Reference	
60‐80 y	1.28 (1.19‐1.38)	<.001	1.21 (1.12‐1.30)	<.001	1.27 (1.14‐1.40)	<.001	1.18 (1.06‐1.31)	.003
>80 y	2.15 (1.96‐2.36)	<.001	1.55 (1.41‐1.71)	<.001	2.22 (1.96‐2.52)	<.001	1.58 (1.37‐1.81)	<.001
Race								
Non‐Hispanic White	Reference				Reference			
Non‐Hispanic Black	0.96 (0.87‐1.05)	.349			0.98 (0.86‐1.11)	.740		
Hispanic	0.95 (0.88‐1.03)	.251			0.98 (0.88‐1.09)	.712		
Others	0.96 (0.86‐1.06)	.372			1.06 (0.93‐1.21)	.366		
Marital status								
Married	Reference		Reference		Reference		Reference	
Divorced/separated	1.11 (1.00‐1.23)	.046	—	.204	1.17 (1.02‐1.34)	.022	1.16 (1.01‐1.33)	.033
Windowed	1.44 (1.33‐1.55)	<.001	—	.397	1.57 (1.42‐1.74)	<.001	1.17 (1.05‐1.31)	.005
Single	1.17 (1.07‐1.28)	.001	—	.255	1.23 (1.08‐1.39)	.001	1.14 (1.01‐1.30)	.039
Histological type								
Adenocarcinoma	Reference		Reference		Reference		Reference	
Squamous cell carcinoma	1.21 (1.03‐1.42)	.019	1.25 (1.06‐1.47)	.007	1.35 (1.11‐1.66)	.003	1.39 (1.13‐1.70)	.002
Others	1.31 (1.22‐1.40)	<.001	1.01 (0.94‐1.09)	.721	1.04 (0.94‐1.15)	.459	0.83 (0.74‐0.92)	.001
Grade								
Grade I	Reference		Reference		Reference		Reference	
Grade II	1.13 (0.04‐1.35)	.204	1.24 (1.03‐1.49)	.022	1.10 (0.87‐1.39)	.446	1.21 (0.96‐1.54)	.105
Grade III	1.64 (1.37‐1.96)	<.001	1.68 (1.41‐2.01)	<.001	1.56 (1.24‐1.96)	<.001	1.66 (1.32‐2.08)	<.001
Grade IV	1.44 (1.09‐1.91)	.010	1.54 (1.16‐2.04)	.003	1.49 (1.05‐2.13)	.027	1.77 (1.24‐2.54)	.002
Unknown	1.85 (1.56‐2.20)	<.001	1.46 (1.22‐1.75)	<.001	1.56 (1.25‐1.95)	<.001	1.38 (1.09‐1.74)	.008
AJCC stage								
III	Reference		Reference		Reference		Reference	
IV	1.40 (1.23‐1.59)	<.001	1.38 (1.21‐1.57)	<.001	1.45 (1.21‐1.72)	<.001	1.44 (1.21‐1.72)	<.001
Treatment pattern								
No surgery/No CT	Reference		Reference		Reference		Reference	
Surgery	0.58 (0.53‐0.63)	<.001	0.60 (0.55‐0.66)	<.001	0.67 (0.60‐0.75)	<.001	0.65 (0.57‐0.74)	<.001
CT	0.41 (0.38‐0.44)	<.001	0.44 (0.40‐0.48)	<.001	0.41 (0.37‐0.46)	<.001	0.44 (0.39‐0.50)	<.001
Surgery + CT	0.26 (0.24‐0.29)	<.001	0.29 (0.26‐0.33)	<.001	0.32 (0.28‐0.36)	<.001	0.33 (0.29‐0.39)	<.001

Abbreviations: AJCC, American Joint Committee on Cancer; CSS, cancer‐specific survival; CT, chemotherapy; Grade I, well differentiated; Grade II, moderately differentiated; Grade III, poorly differentiated; Grade IV, undifferentiated; OS, overall survival.

a Model was adjusted by sex, age, marital status, histological type, grade, AJCC stage, and treatment pattern.

b Model was adjusted by age, marital status, histological type, grade, AJCC stage, and treatment pattern.

**Figure 3:**
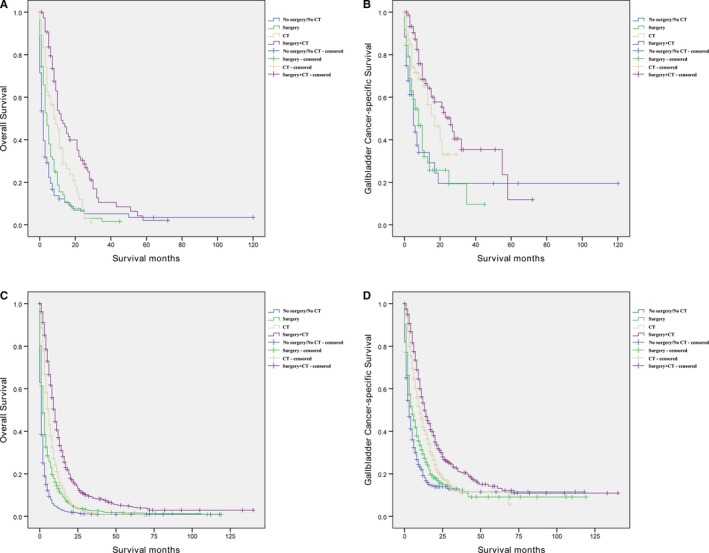
Overall survival and cancer‐specific survival curves of advanced gallbladder cancer patients according to different American Joint Committee on Cancer (AJCC) stage. A and B, Overall survival and cancer‐specific survival of AJCC stage III patients; C and D, Overall survival and cancer‐specific survival of AJCC stage IV patients

To better characterize the influence of treatment pattern on OS and CSS in patients with advanced GBC, we performed AJCC stage stratification on all patient parameters on the basis of multivariate analysis. We found that the treatment pattern was also an independent risk factor for patients with AJCC stage III and AJCC stage IV (Table 4). “Surgery,” “CT,” and “Surgery + CT” improve the AJCC stage III and AJCC stage IV patient OS and CSS. In particular, “Surgery + CT” could significantly improve OS and CSS in both AJCC stage III (HR = 0.36, 95% CI 0.22‐0.60, *P* < .001) and AJCC stage IV (HR = 0.33, 95% CI 0.29‐0.39, *P* < .001) groups (Figure 3).

**Table 4:** Multivariate analysis of OS and CSS rates in AJCC stage III and stage IV before propensity score matching

Characteristic	AJCC stage III (n = 288)				AJCC stage IV (n = 4239)			
	OS^a^		CSS^b^		OS^c^		CSS^d^	
	Hazard ratio (95% CI)	*P* value	Hazard ratio (95% CI)	*P* value	Hazard ratio (95% CI)	*P* value	Hazard ratio (95% CI)	*P* value
Sex								
Female					Reference			
Male					—	.108		
Age at diagnosis								
<60 y	Reference		Reference		Reference		Reference	
60‐80 y	1.10 (0.79‐1.54)	.574	0.97 (0.63‐1.49)	.877	1.22 (1.13‐1.32)	<.001	1.20 (1.08‐1.34)	.001
>80 y	1.99 (1.28‐3.07)	.002	1.92 (1.09‐3.35)	.023	1.54 (1.39‐1.70)	<.001	1.59 (1.38‐1.84)	<.001
Race								
Non‐Hispanic White								
Non‐Hispanic Black								
Hispanic								
Others								
Marital status								
Married	Reference		Reference		Reference		Reference	
Divorced/separated	—	.264	—	.916	—	.240	1.17 (1.02‐1.34)	.030
Windowed	—	.473	—	.006	—	.596	1.14 (1.02‐1.28)	.025
Single	—	.913	—	.750	—	.228	1.15 (1.01‐1.31)	.037
Histological type								
Adenocarcinoma			Reference		Reference		Reference	
Squamous cell carcinoma			1.80 (1.07‐3.05)	.028	—	.029	1.30 (1.04‐1.62)	.021
Others			0.65 (0.40‐1.05)	.076	—	.607	0.84 (0.75‐0.94)	.003
Grade								
Grade I	Reference				Reference		Reference	
Grade II	1.18 (0.64‐2.16)	.598			1.26 (1.04‐1.53)	.018	1.24 (0.97‐1.59)	.090
Grade III	1.80 (0.99‐3.27)	.053			1.71 (1.42‐2.07)	<.001	1.69 (1.33‐2.15)	<.001
Grade IV	1.30 (0.49‐3.48)	.602			1.59 (1.19‐2.14)	.002	1.83 (1.26‐2.66)	.002
Unknown	1.05 (0.56‐1.95)	.888			1.51 (1.25‐1.83)	<.001	1.40 (1.10‐1.79)	.007
Treatment pattern								
No surgery/No CT	Reference		Reference		Reference		Reference	
Surgery	0.69 (0.48‐1.01)	.058	0.72 (0.44‐1.16)	.171	0.60 (0.54‐0.66)	<.001	0.64 (0.56‐0.74)	<.001
CT	0.58 (0.39‐0.87)	.008	0.51 (0.29‐0.90)	.019	0.43 (0.40‐0.47)	<.001	0.44 (0.39‐0.49)	<.001
Surgery + CT	0.32 (0.21‐0.48)	<.001	0.36 (0.22‐0.60)	<.001	0.29 (0.26‐0.33)	<.001	0.33 (0.29‐0.39)	<.001

Abbreviations: AJCC, American Joint Committee on Cancer; CSS, cancer‐specific survival; CT, chemotherapy; Grade I, well differentiated; Grade II, moderately differentiated; Grade III, poorly differentiated; Grade IV, undifferentiated; OS, overall survival.

a Model was adjusted by age, marital status, grade, and treatment pattern.

b Model was adjusted by age, marital status, histological type, and treatment pattern.

c Model was adjusted by sex, age, marital status, histological type, grade, and treatment pattern.

d Model was adjusted by age, marital status, histological type, grade, and treatment pattern.

### 3.4 Identification of prognostic factors of OS and CSS in 1:1 PSM sample

To better balance the patients with AJCC stage III or AJCC stage IV GBC, we performed a 1:1 PSM for variables such as diagnosis age, race, marital status, tumor grade, and treatment pattern to decrease the selection bias and further examine the relationship between treatment patterns and OS and CSS, as assessed with the Cox regression model.

First, we performed univariate and multivariate Cox regression analysis of all patients after PSM, and found that only diagnosis age and treatment pattern were independent risk factors (Table S1). “Surgery + CT” significantly improved the OS (vs “No surgery/No CT”; HR = 0.28, 95% CI 0.21‐0.37, *P* < .001) and CSS (vs “No surgery/No CT”; HR = 0.30, 95% CI 0.22‐0.43, *P* < .001) (Figure S1).

Moreover, we performed AJCC stage stratification on patient parameters after PSM on the basis of multivariate analysis. We found that the treatment pattern was also an independent risk factor for AJCC stage IV patients (Table S2), and “Surgery + CT” significantly improved the OS (vs “No surgery/No CT”; HR = 0.23, 95% CI 0.15‐0.35, *P* < .001) and CSS (vs “No surgery/No CT”; HR = 0.31, 95% CI 0.19‐0.52, *P* < .001) of AJCC stage IV patients after PSM (Figure S2).

## 4 DISCUSSION

Currently, the treatment of patients with advanced GBC remains controversial. Radical surgical resection is the only possible cure treatment for patients with GBC. However, most patients with GBC are at an advanced stage at the time of discovery, thus limiting the opportunity for radical resection; even in the moderate stage, the prognosis of patients undergoing radical resection is highly unsatisfactory. Therefore, clinicians have been exploring the application and combination of adjuvant treatments, including RT, CT, and other treatments, to improve the prognosis of patients with GBC.

Radical surgical resection remains the most important treatment for improving the survival rate of patients with GBC.^13^ Surgeons have long been pessimistic about the treatment of advanced GBC. In recent years, owing to the development of GBC radical surgery, the long‐term survival rate has significantly improved. Nakamura et al^14^ have reported that in 33 GBC patients with Nevin V stage, 13 patients underwent extended radical resection, and the 1‐year and 3‐year survival rates were 46% and 23%, respectively, whereas the 1‐year survival rate of 20 patients without resection was only 15%. Matsumoto et al^15^ reported that the average survival time of 15 patients undergoing extended radical resection was 26 months, whereas that of patients who did not undergo resection was only 10 months.

Patients in stage IV are generally considered unable to undergo surgical resection,^1^ but many clinical studies have supported more aggressive surgical treatment of patients with advanced GBC.^16^, ^17^ Kang's^17^ study has shown that radical surgery in stage IV GBC patients can prolong survival time. Christina et al^18^ have further confirmed this conclusion, suggesting that radical surgery can be performed in stage IV patients as long as the lesion is local and can reach the R0 margin. Studies from Japan also suggest that if the tumor is relatively limited and strictly screened, even if the lesion is large and has invaded adjacent organs, stage IV patients are expected to achieve long‐term survive after radical resection.^19^, ^20^, ^21^, ^22^ However, increased surgical complications and mortality have hampered the adoption of these radical surgical approaches as a standard treatment for GBC.^23^ Similarly, big data research from Japan does not support radical surgical resection in patients in stage IV, and some studies have indicated that radical surgery does not improve prognosis in patients in stage IV.^24^, ^25^

According to the 7th edition of AJCC guidelines, patients with stage T4 are usually considered unresectable and should be treated with palliative care.^26^ Groot et al^27^ have suggested that patients with stage T4 GBC are unlikely to benefit from surgical resection. However, there is currently no consensus regarding the factors indicating unresectable advanced GBC.^28^ Recent reports have shown that radical resection and arteriotomy in advanced GBC, or enlarged right trifoliate resection and hepatopancreatic duodenectomy (HPD) can improve patient prognosis.^29^, ^30^ Nishio et al^31^ have suggested that radical resection also has value for GBC invading the extrahepatic bile duct also. Anil et al^32^ have indicated that even duodenal infiltration of GBC does not indicate that surgical removal is impossible. Our study also shows that the surgery improves OS and CSS in patients with AJCC stage III or AJCC stage IV GBC.

Although surgical radical resection of the GBC is currently extensively performed, the rate of radical resection is only 25%‐30%. After radical resection, nearly half of patients still have a risk of recurrence of GBC. Therefore, to decrease the postoperative recurrence of GBC patients and improve the prognosis of patients with advanced disease, some patients are given CT treatment. At present, gemcitabine combined with oxaliplatin (GEMOX) or cisplatin and tegafur combined with oxaliplatin (SOX) are widely used and recognized as effective chemotherapy regimens for GBC patients.^33^, ^34^

In this large population‐based study, we used the SEER database to analyze the best treatment options for patients with advanced GBC. Through univariate and multivariate Cox survival regression analysis, in all patients, AJCC stage III patients and AJCC stage IV patients, we found that the “Surgery + CT” treatment significantly increase the OS (vs “No surgery/No CT”; HR = 0.29, 95% CI 0.26‐0.33, *P* < .001) and CSS (vs “No surgery/No CT”; HR = 0.33, 95% CI 0.29‐0.39, *P* < .001). The results of 1:1 PSM analysis also showed that the “Surgery + CT” treatment significantly decreased the risk of death in patients with advanced GBC. In addition, the proportion of patients with “Surgery + CT” remained relatively stable over the past 12 years, and “Surgery + CT” may not currently be fully utilized. The combination of surgery and CT may improve the survival rate of patients with advanced GBC.

This study has several limitations. First, it was a retrospective study and thus had clear inherent limitations. Second, the SEER database lacked information about the physical condition and complications of patients, and older patients may be more likely to choose conservative treatment. In addition, the sequence of surgery and CT, as well as the specific regimen of CT was unknown. Nonetheless, the study remains convincing given the large demographics.

## 5 CONCLUSIONS

We found that the “Surgery + CT” treatment model provided greater survival benefits for patients with advanced GBC. Because this was a retrospective analysis, further prospective studies are needed to provide verification.

## AUTHORS' CONTRIBUTIONS

WM, FD, and XS designed the research. WM, FD, and DW performed the research and analyzed results. WM and DW wrote the paper. WM, LG, and XS edited the manuscript and provided critical comments. All authors read and approved the final manuscript.

## COMPLIANCE WITH ETHICAL STANDARDS

### DISCLOSURE OF POTENTIAL CONFLICT OF INTEREST

We declare that there is no conflict of interest between authors.

### RESEARCH INVOLVING HUMAN PARTICIPANTS AND/OR ANIMALS

This article does not contain any studies with human participants or animals performed by any of the authors.
