# Supplementary figures and images for: Treatment of advanced gallbladder cancer: A SEER‐based study
Source: Cancer Med. 2019 Nov 13;9(1):141–50. doi: 10.1002/cam4.2679 (PMC6943088; doi:10.1002/cam4.2679)

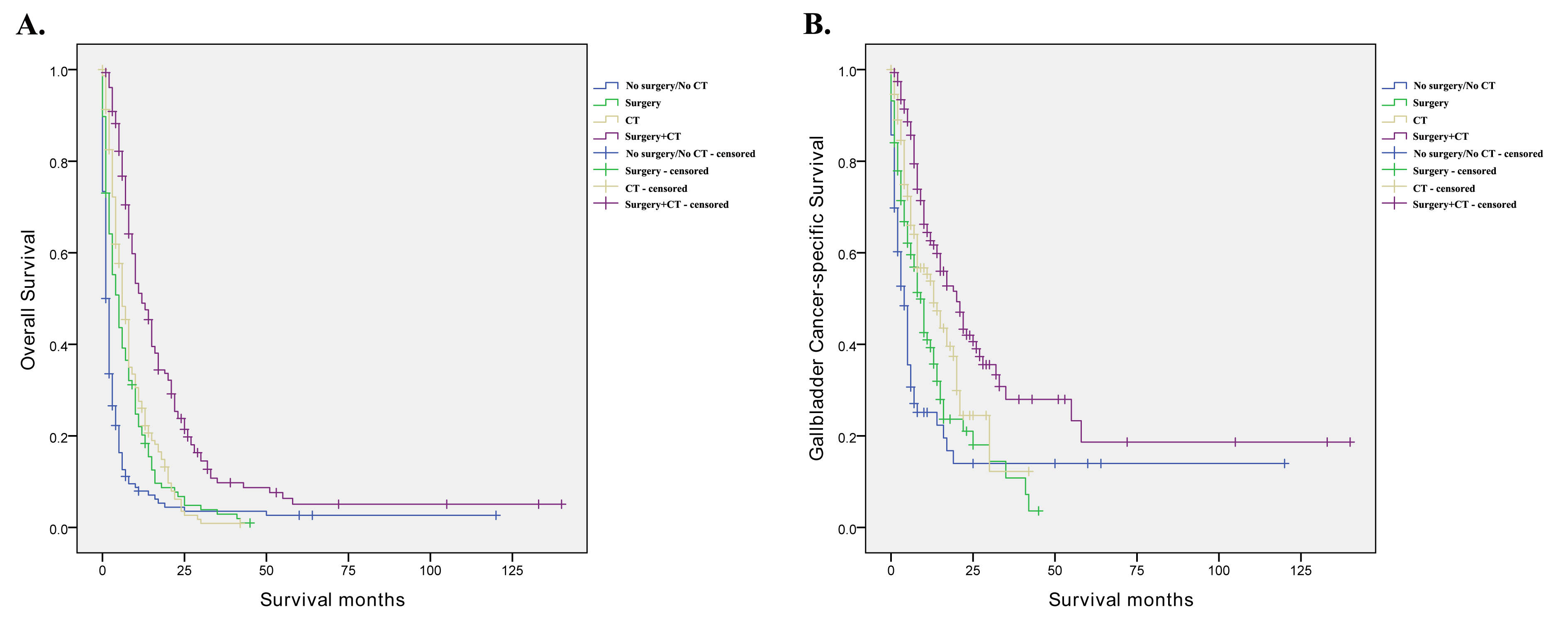

Supplement: Supplementary file 1 [file CAM4-9-141-s001.tif]

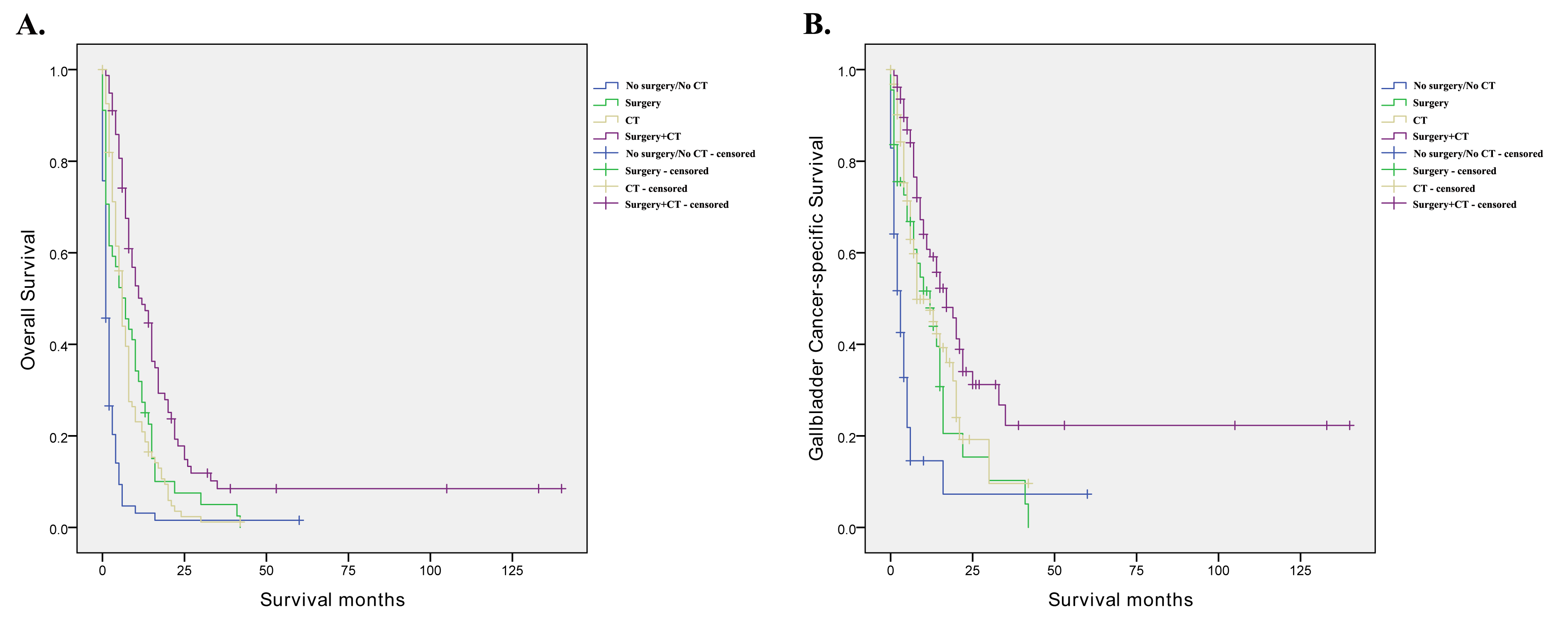

Supplement: Supplementary file 2 [file CAM4-9-141-s002.tif]
