# Supplementary material for: Treatment of advanced gallbladder cancer: A SEER‐based study
Source: Cancer Med. 2019 Nov 13;9(1):141–50. doi: 10.1002/cam4.2679 (PMC6943088; doi:10.1002/cam4.2679)
Supplement: Supplementary file 3 [file CAM4-9-141-s003.docx]

**Table S1. Univariate and multivariate analysis of overall survival (OS) and cancer-specific survival (CSS) rates in the 1:1 propensity score matching sample.**

| **Characteristic** | **OS** | | | | **CSS** | | | |
| --- | --- | --- | --- | --- | --- | --- | --- | --- |
|  | **Univariate analysis** | | **Multivariate analysis^a^** | | **Univariate analysis** | | **Multivariate analysis^b^** | |
|  | **Hazard Ratio (95% CI)** | **P value** | **Hazard Ratio (95% CI)** | **P value** | **Hazard Ratio (95% CI)** | **P value** | **Hazard Ratio (95% CI)** | **P value** |
| Sex |  |  |  |  |  |  |  |  |
| Female | Reference |  |  |  | Reference |  |  |  |
| Male | 0.92 (0.76-1.11) | 0.399 |  |  | 0.84 (0.66-1.07) | 0.160 |  |  |
| Age at diagnosis |  |  |  |  |  |  |  |  |
| < 60 years | Reference |  | Reference |  | Reference |  | Reference |  |
| 60-80 years | 1.04 (0.87-1.26) | 0.661 | 0.93 (0.77-1.13) | 0.473 | 0.97 (0.76-1.25) | 0.831 | 0.85 (0.66-1.10) | 0.210 |
| > 80 years | 2.51 (1.91-3.32) | <0.001 | 1.55 (1.14-2.10) | 0.005 | 2.41 ()1.67-3.48 | <0.001 | 1.41 (0.95-2.11) | 0.090 |
| Race |  |  |  |  |  |  |  |  |
| Non-Hispanic White | Reference |  |  |  | Reference |  | Reference |  |
| Non-Hispanic Black | 1.32 (1.01-1.71) | 0.040 |  |  | 1.46 (1.04-2.04) | 0.028 | - | 0.081 |
| Hispanic | 0.91 (0.68-1.21) | 0.508 |  |  | 0.84 (0.56-1.26) | 0.394 | - | 0.197 |
| Others | 1.18 (0.93-1.49) | 0.174 |  |  | 1.36 (1.01-1.83) | 0.045 | - | 0.075 |
| Marital status |  |  |  |  |  |  |  |  |
| Married | Reference |  | Reference |  | Reference |  | Reference |  |
| Divorced/Separated | 1.28 (0.98-1.68) | 0.071 | - | 0.201 | 1.19 (0.82-1.71) | 0.357 | - | 0.555 |
| Windowed | 1.44 (1.10-1.88) | 0.008 | - | 0.908 | 1.71 (1.23-2.39) | 0.001 | - | 0.122 |
| Single | 1.15 (0.88-1.52) | 0.307 | - | 0.405 | 1.07 (0.73-1.56) | 0.743 | - | 0.245 |
| Histological type |  |  |  |  |  |  |  |  |
| Adenocarcinoma | Reference |  |  |  | Reference |  |  |  |
| Squamous cell carcinoma | 1.09 (0.76-1.56) | 0.643 |  |  | 1.41 (0.93-2.13) | 0.107 |  |  |
| Others | 1.18 (0.96-1.47) | 0.119 |  |  | 0.96 (0.71-1.29) | 0.793 |  |  |
| Grade |  |  |  |  |  |  |  |  |
| Grade I | Reference |  | Reference |  | Reference |  | Reference |  |
| Grade II | 1.05 (0.66-1.66) | 0.845 | 1.08 (0.69-1.72) | 0.731 | 0.82 (0.47-1.42) | 0.474 | - | 0.068 |
| Grade III | 1.59 (1.02-2.49) | 0.043 | 1.66 (1.06-2.61) | 0.028 | 1.23 (0.72-2.10) | 0.442 | - | 0.007 |
| Grade IV | 0.88 (0.41-1.86) | 0.732 | 0.99 (0.47-2.09) | 0.974 | 0.85 (0.35-2.09) | 0.729 | - | 0.851 |
| Unknown | 1.72 (1.11-2.67) | 0.016 | 1.20 (0.75-1.91) | 0.457 | 1.29 (0.76-2.17) | 0.345 | - | 0.463 |
| AJCC stage |  |  |  |  |  |  |  |  |
| III | Reference |  |  |  | Reference |  |  |  |
| IV | 1.08 (0.91-1.29) | 0.367 |  |  | 1.21 (0.97-1.53) | 0.098 |  |  |
| Treatment pattern |  |  |  |  |  |  |  |  |
| No surgery/No CT | Reference |  | Reference |  | Reference |  | Reference |  |
| Surgery | 0.56 (0.44-0.72) | <0.001 | 0.57 (0.43-0.77) | <0.001 | 0.61 (0.44-0.85) | 0.003 | 0.63 (0.45-0.88) | 0.006 |
| CT | 0.47 (0.37-0.60) | <0.001 | 0.51 (0.39-0.66) | <0.001 | 0.42 (0.31-0.59) | <0.001 | 0.45 (0.31-0.64) | <0.001 |
| Surgery+CT | 0.27 (0.21-0.34) | <0.001 | 0.28 (0.21-0.37) | <0.001 | 0.28 (0.21-0.39) | <0.001 | 0.30 (0.22-0.43) | <0.001 |

Abbreviations:

OS, Overall survival; CSS, Cancer-specific survival; AJCC, American Joint Committee on Cancer; CT, Chemotherapy.

Grade I, Well differentiated; Grade II, Moderately differentiated; Grade III, Poorly differentiated; Grade IV, Undifferentiated.

^a^Model was adjusted by age, marital status, grade and treatment pattern.

^b^Model was adjusted by age, race, marital status, grade and treatment pattern.

**Table S2. Multivariate analysis of overall survival (OS) and cancer-specific survival (CSS) rates in AJCC stage III and stage IV in the 1:1 propensity score matching sample.**

| **Characteristic** | **AJCC stage III (n = 288)** | | | | **AJCC stage IV (n = 288)** | | | |
| --- | --- | --- | --- | --- | --- | --- | --- | --- |
|  | **OS^a^** | | **CSS^b^** | | **OS^c^** | | **CSS^d^** | |
|  | **Hazard Ratio (95% CI)** | **P value** | **Hazard Ratio (95% CI)** | **P value** | **Hazard Ratio (95% CI)** | **P value** | **Hazard Ratio (95% CI)** | **P value** |
| Sex |  |  |  |  |  |  |  |  |
| Female |  |  |  |  |  |  |  |  |
| Male |  |  |  |  |  |  |  |  |
| Age at diagnosis |  |  |  |  |  |  |  |  |
| < 60 years | Reference |  | Reference |  | Reference |  | Reference |  |
| 60-80 years | 1.10 (0.79-1.54) | 0.574 | 0.97 (0.63-1.49) | 0.877 | - | 0.590 | - | 0.703 |
| > 80 years | 1.99 (1.28-3.07) | 0.002 | 1.92 (1.09-3.35) | 0.023 | - | 0.372 | - | 0.064 |
| Race |  |  |  |  |  |  |  |  |
| Non-Hispanic White |  |  |  |  |  |  |  |  |
| Non-Hispanic Black |  |  |  |  |  |  |  |  |
| Hispanic |  |  |  |  |  |  |  |  |
| Others |  |  |  |  |  |  |  |  |
| Marital status |  |  |  |  |  |  |  |  |
| Married | Reference |  | Reference |  |  |  |  |  |
| Divorced/Separated | - | 0.264 | - | 0.916 |  |  |  |  |
| Windowed | - | 0.473 | - | 0.006 |  |  |  |  |
| Single | - | 0.913 | - | 0.750 |  |  |  |  |
| Histological type |  |  |  |  |  |  |  |  |
| Adenocarcinoma |  |  | Reference |  | Reference |  | Reference |  |
| Squamous cell carcinoma |  |  | 1.80 (1.07-3.05) | 0.028 | 2.20 (1.24-3.91) | 0.007 | 2.56 (1.28-5.13) | 0.008 |
| Others |  |  | 0.65 (0.40-1.05) | 0.076 | 1.19 (0.86-1.67) | 0.299 | 1.05 (0.66-1.68) | 0.825 |
| Grade |  |  |  |  |  |  |  |  |
| Grade I | Reference |  |  |  | Reference |  |  |  |
| Grade II | 1.18 (0.64-2.16) | 0.598 |  |  | 2.10 (1.19-3.70) | 0.010 |  |  |
| Grade III | 1.80 (0.99-3.27) | 0.053 |  |  | 2.73 (1.58-4.70) | <0.001 |  |  |
| Grade IV | 1.30 (0.49-3.48) | 0.602 |  |  | 1.84 (0.53-6.38) | 0.335 |  |  |
| Unknown | 1.05 (0.56-1.95) | 0.888 |  |  | 1.70 (0.99-2.92) | 0.054 |  |  |
| Treatment pattern |  |  |  |  |  |  |  |  |
| No surgery/No CT | Reference |  | Reference |  | Reference |  | Reference |  |
| Surgery | 0.69 (0.48-1.01) | 0.058 | 0.72 (0.44-1.16) | 0.171 | 0.50 (0.33-0.76) | 0.001 | 0.69 (0.42-1.14) | 0.151 |
| CT | 0.58 (0.39-0.87) | 0.008 | 0.51 (0.29-0.90) | 0.019 | 0.45 (0.31-0.66) | <0.001 | 0.32 (0.19-0.55) | <0.001 |
| Surgery+CT | 0.32 (0.21-0.48) | <0.001 | 0.36 (0.22-0.60) | <0.001 | 0.23 (0.15-0.35) | <0.001 | 0.31 (0.19-0.52) | <0.001 |

Abbreviations:

OS, Overall survival; CSS, Cancer-specific survival; AJCC, American Joint Committee on Cancer; CT, Chemotherapy.

Grade I, Well differentiated; Grade II, Moderately differentiated; Grade III, Poorly differentiated; Grade IV, Undifferentiated.

^a^Model was adjusted by age, marital status, grade, and treatment pattern.

^b^Model was adjusted by age, marital status, histological type, and treatment pattern.

^c^Model was adjusted by age, histological type, grade, and treatment pattern.

^d^Model was adjusted by age, histological type, and treatment pattern.
